# Identification of Arbuscular Mycorrhiza (AM)-Responsive microRNAs in Tomato

**DOI:** 10.3389/fpls.2016.00429

**Published:** 2016-03-31

**Authors:** Ping Wu, Yue Wu, Cheng-Chen Liu, Li-Wei Liu, Fang-Fang Ma, Xiao-Yi Wu, Mian Wu, Yue-Yu Hang, Jian-Qun Chen, Zhu-Qing Shao, Bin Wang

**Affiliations:** ^1^State Key Laboratory of Pharmaceutical Biotechnology, Laboratory of Plant Genetics and Molecular Evolution, School of Life Sciences, Nanjing UniversityNanjing, China; ^2^Institute of Botany, Jiangsu Province and Chinese Academy of SciencesNanjing, China

**Keywords:** arbuscular mycorrhiza symbiosis, MicroRNAs, *Rhizophagus irregularis*, tomato, deep sequencing analysis

## Abstract

A majority of land plants can form symbiosis with arbuscular mycorrhizal (AM) fungi. MicroRNAs (miRNAs) have been implicated to regulate this process in legumes, but their involvement in non-legume species is largely unknown. In this study, by performing deep sequencing of sRNA libraries in tomato roots and comparing with tomato genome, a total of 700 potential miRNAs were predicted, among them, 187 are known plant miRNAs that have been previously deposited in miRBase. Unlike the profiles in other plants such as rice and *Arabidopsis*, a large proportion of predicted tomato miRNAs was 24 nt in length. A similar pattern was observed in the potato genome but not in tobacco, indicating a Solanum genus-specific expansion of 24-nt miRNAs. About 40% identified tomato miRNAs showed significantly altered expressions upon *Rhizophagus irregularis* inoculation, suggesting the potential roles of these novel miRNAs in AM symbiosis. The differential expression of five known and six novel miRNAs were further validated using qPCR analysis. Interestingly, three up-regulated known tomato miRNAs belong to a known miR171 family, a member of which has been reported in *Medicago truncatula* to regulate AM symbiosis. Thus, the miR171 family likely regulates AM symbiosis conservatively across different plant lineages. More than 1000 genes targeted by potential AM-responsive miRNAs were provided and their roles in AM symbiosis are worth further exploring.

## Introduction

Plants can interact with varied microbes to form mutual relationships, which assists the absorption of nutrients including nitrogen, phosphates, and metal elements from the soil (Oldroyd, 2013). The two most intensively studied plant-microbe symbioses are plant-rhizobium symbiosis and plant-arbuscular mycorrhiza (AM) fungi symbiosis (Oldroyd, 2013). While symbioses with rhizobia are mainly restricted to legume plants, the associations with AM fungi have existed for over 80% of the land plants (Wang and Qiu, 2006). The AM symbiosis, therefore, represents one of the most ancient and significant plant-microbe symbioses that facilitate plant colonization of the terrestrial environment (Pirozynski and Malloch, 1975; Wu et al., 2010b). In this process, the plants supply nutrition (carbohydrates) to the AM fungi while AM fungi improve the water and nutrient (mainly phosphorus) uptake of plants.

Several studies have identified the molecular components from both partners controlling the establishment and maintenance of plant-microbe symbioses (Delaux et al., 2013; Oldroyd, 2013; Schmitz and Harrison, 2014). Plant roots exude strigolactones during symbiosis to induce AM fungi spore germination and hyphal branching (Oldroyd, 2013; Schmitz and Harrison, 2014). Then, the germinated AM fungi release Myc factors (perceived by a plant membrane-anchored receptors) that subsequently activate the downstream factors known as DMI proteins, NSP1, NSP2, RAM1, and RAM2 in plant cells (Delaux et al., 2014; Schmitz and Harrison, 2014). Several of these downstream-activated factors are essential for both bacterial and fungal colonization and belong to a common symbiotic pathway (Oldroyd, 2013; Schmitz and Harrison, 2014). Activation of the common symbiotic pathway leads to the establishment of symbiosis through a transcriptional reprogramming of dozens to thousands of genes (Guether et al., 2009; Xue et al., 2015). Yet, the molecular mechanisms of plant-microbe symbiosis have not been fully decoded, and new factors involved in this process continue to be identified (Schmitz and Harrison, 2014; Venkateshwaran et al., 2015).

Plant microRNAs (miRNAs), a class of small non-coding RNAs that are usually 21–24 nucleotides (nt) in length, function to regulate gene expression at transcriptional and post-transcriptional levels by loading into AGO proteins and forming the RISC (RNA-induced silencing complex; Jones-Rhoades et al., 2006). Most plant miRNA precursors (pre-miRNAs) are generally transcribed by RNA polymerase II (Pol II) and processed by DICER-LIKE1 (DCL1) to generate miRNAs that are predominantly 21 nt in length (Rogers and Chen, 2013). Plants also have another miRNA process path, in which 24 nt long-miRNAs are generated depending on DCL3 and can be distinguished from siRNAs by their independence of RDR2 (Vazquez et al., 2008; Chellappan et al., 2010; Wu et al., 2010a).

Since the first plant miRNA was identified in 2002 (Llave et al., 2002a), a great number of miRNAs have been identified from various species. Functional studies also characterized a collection of miRNAs that play important roles in plant developmental processes, hormonal signaling, organogenesis, and resistance to abiotic and biotic stresses (Yang and Huang, 2014; Li and Zhang, 2016). Several studies revealed that miRNAs also play roles in plant-microbe symbiosis. For example, miR166 and miR169 regulate nodule organogenesis in *Medicago trunctula* (Combier et al., 2006; Boualem et al., 2008). MiR171 and miR397 are associated with nodule infection and the nitrogen-fixing ability of *Lotus japonicus* (De Luis et al., 2012). Two recent studies independently found that miR172c modulates the rhizobium infection and nodule organization in both soybean and *Lotus japonicus* by targeting the transcription factor AP2 (Wang et al., 2014; Holt et al., 2015). Plant miRNAs involved in AM symbiosis have also been reported. Branscheid et al. (2010) first reported the accumulation of miR399 in the roots of *M. trunctula* and tobacco (*Nicotiana tabacum*) during AM symbiosis (Branscheid et al., 2010). Through deep sequencing analysis, they further identified eight *M. trunctula* miRNA families that show strong expression alteration during AM symbiosis (Devers et al., 2011). Two following studies then revealed that *M. trunctula* miR396 and miR171h regulate root architecture and symbiosis with AM fungi by respectively targeting growth-regulating factor gene (*MtGRF*) and Nodulation Signaling Pathway2 (*NSP*2) gene (Lauressergues et al., 2012; Bazin et al., 2013).

Despite the recent progresses in discovering symbiosis-related miRNAs in legumes, few studies explore the cases in non-legume plants. This hinders the identification of potential conserved miRNAs in AM symbiosis across different plant lineages and uncovering novel lineage-specific AM-responsive miRNAs. Tomato is a non-legume plant and is often used for AM symbiosis studies. Several previous studies have surveyed miRNA composition of various tomato tissues through traditional cloning, high throughput sequencing or genome-wide prediction (Pilcher et al., 2007; Itaya et al., 2008; Moxon et al., 2008), but few of them have focused on tomato roots under AM symbiotic condition through deep sequencing (Karlova et al., 2013; Din and Barozai, 2014). Through microarray screening, Gu et al. have found that sly-miR158, sly-miR399h, sly-miR408a, sly-miR827b, and sly-miR399m were differentially expressed in tomato root during AM symbiosis (Gu et al., 2010, 2014). In order to uncover the miRNA profile in tomato root and explore more AM-responsive miRNAs, we used high-throughput technology to sequence the sRNA libraries in tomato roots colonized or not colonized by *Rhizophagus irregularis* and identified a profile of 700 potential miRNAs. While 187 of them belong to known miRNA families in miRBase, the rest 513 are largely newly reported. Our study will provide insight into the miRNA profile of tomato roots and help in exploring their target genes that potentially involved in AM symbiosis.

## Materials and methods

### Plant materials

Seeds of tomato (*Solanum lycopersicum*) were surface sterilized with 8% sodium hypochlorite for 6 min by gently shaking, and rinsed six times in sterile distilled water. Surface sterilized tomato seed were germinated at 24°C for 2 days on 0.8% water Agar. After germinating, the seeds were transferred to a plastic pot (12 cm upper diameter × 11 cm height × 8 cm bottom diameter), each filled with 650 g sands, which were autoclaved at 121°C for 2 h after passing through a 2 mm sieve. For mycorrhizal treatment, plants were inoculated with *R. irregularis* (BGC HEB07D, a mixture of soil, spores, hyphae, and root material from the Institute of Plant Nutrition and Resources, Beijing Academy of Agriculture and Forestry Sciences, China) while the control sample received the same amount of autoclaved inoculum. Plants were cultivated under 16 h light (300 μmol photons m^−2^ s^−1^) at 24°C and 8 h dark at 22°C. All plants were watered two times per week with half-strength Hoagland solution containing 20 μM phosphates. We harvested the plants 75 days after inoculation. The mycorrhizal establishment was confirmed by morphological assessment (stained by black ink) and detecting the expression of an AM symbiosis marker gene, *SlPT4*.

### RNA extraction, small RNA library construction, and sequencing

Total RNA from tomato roots of the control and treated (inoculated with *R. irregularis*) samples used in this study were isolated using TRIzol reagent (Invitrogen, Carlsbad, CA, USA) according to the manufacture's protocol. The RNA integrity was examined on Agilent 2100. Small RNAs (sRNAs) of 18-30 nt were separated by 15% PAGE. After ligation to a pair of adaptors to the 5′ and 3′ ends, sRNAs were reversed transcribed to cDNA and amplified using PCR and finally sequenced at Beijing Genomics Institute (BGI, Shenzhen, China). The results were deposited in the Sequence Read Archive (SRA) at the NCBI database (accession number: GSE76204).

### Bioinformatics prediction of known miRNAs and novel miRNAs

After the sequencing reactions were complete, we trimmed the adaptor sequences and removed the low-quality tags, the sequences less than 18 nt, and the sequences with null adaptors. Small RNAs that matched to rRNAs, tRNAs and other noncoding RNA with no more than 2 mismatches were removed. The reads corresponding to the exon and repeat sequences were excluded. The remaining reads that could be mapped to the *Solanum lycopersicum* genome were aligned with all plant miRNAs in the miRBase 21.0 database (http://www.mirbase.org/) to identify the conserved miRNAs using bowtie. Then the unannotated reads were analyzed for prediction of novel miRNAs using the program mireap (developed by BGI, https://sourceforge.net/projects/mireap/). The parameters for miRNAs prediction are as follows: (1) minimal miRNA sequence length (18); (2) maximal miRNA sequence length (25); (3) minimal miRNA reference sequence length (20); (4) maximal miRNA reference sequence length (23); (5) maximal copy number of miRNAs on reference (20); (6) maximal free energy allowed for a miRNA precursor (−18 kcal/mol); (7) maximal space between miRNA and miRNA* (200); (8) minimal base pairs of miRNA and miRNA* (16); (9) maximal bulge of miRNA and miRNA* (4); (10) maximal asymmetry of miRNA/miRNA* duplex (4); (11) flank sequence length of miRNA precursor (25). A minimum abundance of 10 reads in at least one library was set as a common criterion for the identification of both known and novel miRNAs in this study.

### Identification of AM symbiosis responsive miRNAs

To identify AM symbiosis responsive miRNAs, we normalized the expressions of miRNAs to 1 million (RPM) in each sample (actual miRNA count/total count of clean data × 10^6^). The expression level of each miRNA between the control and treatment was analyzed as described in a previous study (Zhang et al., 2014). The miRNAs with fold-change > 2 or < −2 and *P* < 0.001 were considered as AM symbiosis responsive miRNAs. The fold-change was calculated using the formula: fold-change = log2 (treatment/control). The *P*-value was calculated according to the methods described by Man et al. (2000).

### Identification of miRNA targets

The putative target genes were predicted by the psRNATarget algorithm (http://plantgrn.noble.org/psRNATarget/). The prediction rules were as follows: (1) Maximum expectation is less than 3.0. (2) Length for complementarity scoring (hspsize) is shorter than 20. (3)Target accessibility - allowed maximum energy to unpair the target site (UPE) is shorter than 25. (4) Flanking length around target site for target accessibility analysis is 17 nt in the upstream and 13 in the downstream. (5) Range of central mismatch leading to translational inhibition is 9–11 nt. The mature sequences of known miRNAs and novel miRNAs were submitted to predict target genes against the library (*S. lycopersicum* (tomato), transcript, cDNA library, version 2.4). The predicted miRNA-mediated RNA cleavage events were further verified by searching the degradome data sets through the web server StarScan (Liu et al., 2015). To elucidate the potentially related biological processes and pathways involved in symbiosis, the predicted target genes were annotated by Gene Ontology (http://www.Geneontology.org/) and KEGG pathway (http://www.genome.jp/kaas-bin/kaas_main).

### *PHAS* loci identification

Our method is similar to the previous methods used for PhasiRNA identification (De Paoli et al., 2009; Zheng et al., 2015). We denoted the sRNA sequences after mapping to the genome. Two-nucleotide positive offsets were added to the 3′ end overhand when matching sRNAs to the antisense strand of the genome. The search was conducted using ten-phase slide window (210 nt). *P*-value was calculated for each window based on the following formulas.

$$\begin{array}{l} r\left(X=k\right)=\frac{\left(\begin{array}{c} 20m\\ n-k \end{array}\right)\left(\begin{array}{c} m\\ k \end{array}\right)}{\left(\begin{array}{c} 21m\\ n \end{array}\right)} \end{array}$$

*P*-value: $p\left(k\right)=\sum_{X=k}^{m}Pr\left(X\right)$ where n was the total number of unique 21-nt reads matched in a window, *k* was the number of unique 21-nt reads matched to a phased position in a window, and m was the number of 21-nt phase in a window. Window with a *P*-value less than 0.001 was positive.

Phasing score was calculated using previous method (De Paoli et al., 2009), with a ten-cycle window (210 nt).

$$\begin{array}{l} phasing\;score=ln\left[{\left(1+10\times \frac{\sum_{i=1}^{10}P_{i}}{1+\sum U}\right)}^{k-2}\right],k>3 \end{array}$$

Where k was the number of phases occupied by at least one 21-nt reads within the window, P was the total number of 21-nt reads falling into a phase in a window, and U was the total number of reads out of phase in the window.

### Validation of the expression of miRNAs and *SlPT4* gene by RT-qPCR

Expression of the miRNAs and *SlPT4* gene was validated by RT-qPCR. Total RNA was treated with DNase (QIAGEN), and then cDNA synthesis was performed following the manufacturer's protocol using the mir-X miRNA first-strand synthesis kit (Takara, Japan) or SuperScript™ III First-Strand Synthesis System (Invitrogen). qPCR was performed using the CFX96 real-time system (Bio-Rad) or StepOnePlus real-time PCR system (Applied Biosystems) with SYBR Green PCR master mix (Takara, Japan). In brief, 1 μl cDNA were added to 10 μl SYBR Green PCR master mix, 0.5 μl 10 μM Primers and ddH_2_O to a final volume of 20 μl. The amplification reaction was pre-degenerated for 30 s at 95.0°C, followed by 40 cycles of 95.0°C for 10 s and 60.0°C for 30 s. All the reactions were performed in triplicate. Primers used for amplifying are listed in the Additional file: Supplementary Table S5.

## Results

### Deep sequencing and analysis of sRNAs in tomato roots

Two separate sRNA libraries were constructed and sequenced from tomato roots treated with and without *R. irregularis* respectively after confirming the formation of AM symbiosis (Supplementary Figure S1). A total of 14,191,678 raw reads from the treatment library and 14,231,261 reads from the control library were first collected. After removing the adaptor and filtering out low-quality reads, null and short sequences, 13,928,894 high-quality, clean reads in the treatment sample and 14,005,887 such reads in the control sample were obtained for further analysis (Table 1). Among them, 71.35% were common to both libraries, while 12.45 and 16.20% reads were specific to the treatment and the control libraries, respectively (Supplementary Figure S2). In length, a majority (>80%) of these small RNA read sequences from the two libraries were 20–24 nt and the most abundant were 24 nt in length, followed by 21 and 22 nt (Supplementary Figure S2).

**Table 1:** **Statistics of sequencing reads for two libraries from tomato**.

**Type**	**Treatment**	**Control**
Total raw reads	14,191,678	14,231,261
High quality reads	14,087,846	14,124,609
3′adapter_null	14,034 (0.10%)	13,337 (0.09%)
insert_null	2151 (0.02%)	1603 (0.01%)
5′adapter_contaminants	34,151 (0.24%)	19,282 (0.14%)
polyA	1717 (0.01%)	1236 (0.01%)
Reads smaller than 18nt	106,899 (0.76%)	83,264 (0.5%)
Clean reads	13,928,894 (98.87%)	14,005,887 (99.16%)

The obtained high-quality, clean read sequences can be merged into a total of 4,621,745 unique sequences for the treatment sample and 4.039,655 unique sequences for the control sample. The size distribution pattern of the unique reads was different from that of total reads, with 24 and 23 nt being the two predominant size classes in both libraries (Supplementary Figure S2). About 65.96% (3,048,316) unique sequences in the treatment sample and 66.84% (2,700,260) unique sequences in the control sample can align to the tomato genome. Among them, 4535 and 3421 unique reads in the two libraries were annotated as tRNA, rRNA or other non-coding RNAs (Table 2). Moreover, about 1,000,000 clean reads in the treatment and control samples matched to exon or repeat regions (Table 2). In the remaining reads, 1239 treatment reads and 1043 control reads can match to known miRNAs in miRBase, respectively; while the rest 1,974,401 treatment reads and 1,721,502 control reads match to non-coding regions of tomato genome and likely harbor novel miRNAs (Table 2).

**Table 2:** **Categorization of unique reads of small RNAs in two libraries**.

**Category**	**Treatment**	**Control**
Clean reads	4,621,745	4,039,655
Map to tomato genome	3,048,316	2,700,260
Rfam^a^	4535	3421
Repeat	913,523	815,672
Exon	154,618	158,622
miRBase	1239	1043
Unannotated	1,974,401	1,721,502

a *Rfam, reads mapped to plant rRNA, tRNA, snoRNA in Rfam*.

### Identification of known plant miRNAs in tomato roots

**Figure 1:**
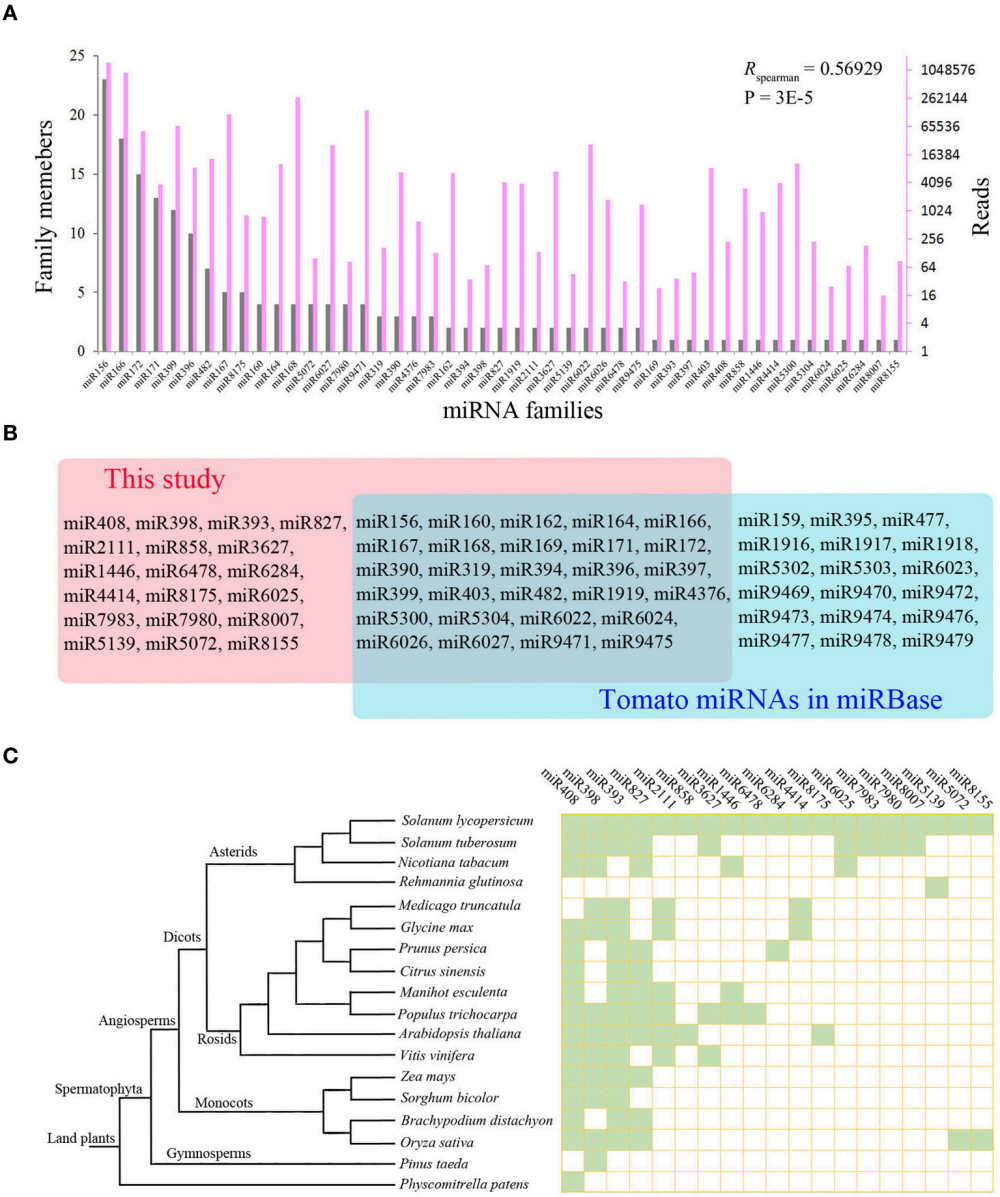
**Known miRNAs identified from tomato root. (A)** The correlation of family members and the abundance of miRNA families. **(B)** Known miRNAs predicted in tomato root. The pink rectangle represents known miRNAs that have not been document for tomato in the miRBase, and the blue rectangle represents known tomato miRNAs in miRBase. **(C)** Phylogenetic distribution of known miRNAs in tomato identified in this study.

The clean read sequences matched to known miRNAs in miRBase were further screened to discard low expression ones with fewer than 10 reads in both libraries, and a total of 187 known miRNAs belonging to 47 miRNA families (Supplementary Table S1) were finally kept from the two libraries. The number of family members varied greatly among the 47 miRNA families (Figure 1A). Five miRNA families (miR399, miR156, miR166, miR171, and miR172) had more than 10 members, and miR156 family, the largest family, had 23 members. In contrast, several miRNA families such as miR403, miR408, miR6284, miR6025, and miR6024 have only one member. The reads number for these known miRNAs also varied to a large extent ranging from 1 to 363294, with miR166, miR156, and miR168 families having the most abundant reads in the two libraries. Furthermore, a significant positive correlation was observed between the family members and the number of reads in the 47 miRNA families. Classification of known miRNAs according to their sequence length revealed that 21-nt miRNAs were the major type accounting for 118 of all miRNAs.

Previously in tomato, 110 known miRNAs belonging to 46 miRNA families were identified and documented in the miRBase. Twenty eight of these families were detected in our data, in addition to the other 19 known miRNA families, which have not been document for tomato in the miRBase (Figure 1B). Searching homologous sequences from other plant species in the miRBase revealed that four of the 19 miRNA families (miR408, miR398, miR393, and miR827) were ancient miRNA families, and their family members had been identified in more than 10 different plant species. In contrast, another four miRNA families (miR6025, miR7980, miR7983, and miR8007) were only found in *Solanaceae* species indicating their recent emergence (Figure 1C). By searching other studies for tomato miRNAs, we found that 10 of these 19 miRNAs have also been reported in independent studies (Karlova et al., 2013; Luan et al., 2014; Gao et al., 2015), suggesting these are likely true miRNAs in tomato and should be included in the miRBase. The other nine known miRNA families were first identified in tomato, including miR3627, miR4414, miR5072, miR5139, miR6284, miR6478, miR7980, miR8155, and miR8175.

### Identification of novel miRNAs in tomato roots

**Figure 2:**
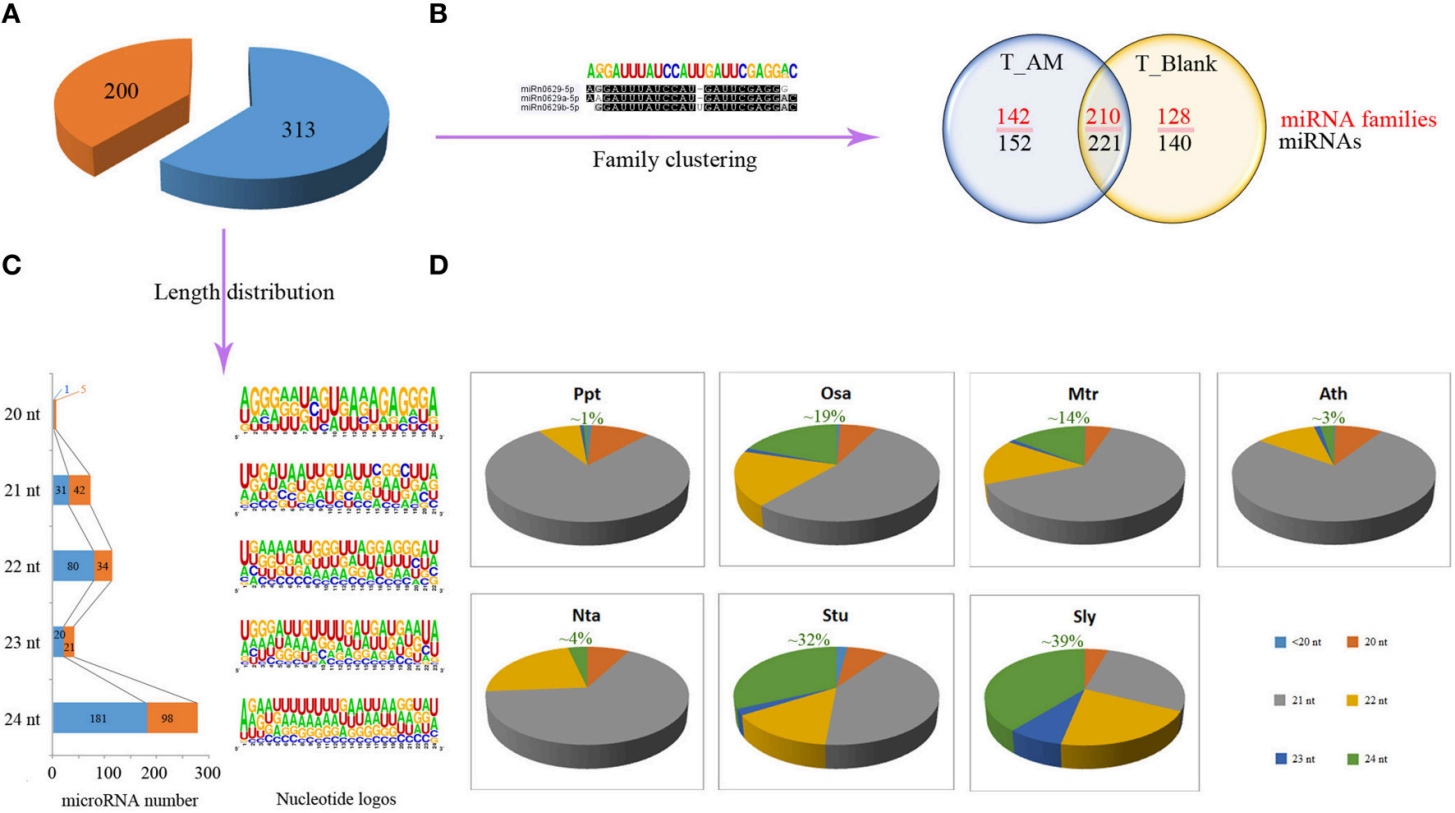
**Characters of novel miRNAs in tomato. (A)** 513 Novel miRNAs were identified in tomato root, 200 of which have detected miRNA stars. **(B)** Novel miRNAs were clustered into 480 families based on Sequence identity cut-off 0.85. Their distribution in the two libraries was shown. **(C)** Sequence logos for novel miRNAs of different lengths. **(D)** Length distribution of miRNAs in *Physcomitrella patens* (Ppt)*, Oryza sativa* (Osa)*, Medicago trunc*a*tula* (Mtr)*, Arabidopsis thaliana* (Ath)*, Nicotiana tabacum* (Nta)*, Solanum tuberosum* (Stu), and *Solanum lycopersicum* (Sly).

Using mireap software with optimized parameters for plant miRNA prediction, the rest reads from the treatment (1,974,401) and control (1,721,502) libraries that match to non-coding regions of tomato genome were screened to identify potential novel miRNAs, which show hairpin structures in their precursors. Five hundred and thirteen novel miRNAs were predicted, of which 200 even had detected miRNA stars (Figure 2A). The predicted miRNAs with detected stars are more likely bona fide novel miRNAs, whereas other predicted miRNAs are potential novel miRNAs. The predicted length of the novel miRNA precursors ranges from 68 to 293 nt, with their minimum folding free energies ranging from −18 to −183.1 kcal/mol (Supplementary Table S2). Using a cutoff of 85% identity, these miRNAs were further clustered into 480 families, among which 210 were presented in both libraries while 142 and 128 were specific to the treatment and control libraries respectively (Figure 2B). All identified novel miRNAs were 20–24 nt in length, and 55% of them were 24-nt miRNAs, representing the most abundant miRNA type in tomato. Further analysis of the 200 bona fide novel miRNAs showed that 49% of them are 24 nt. We also analyzed the nucleotide bias of novel miRNAs in different lengths. As shown in Figure 2C, short miRNAs often have a U at the 5′ first position. In contrast, the 24-nt miRNAs tend to have an A at the 5′ first position. This is in accordance with findings in other plants (Jain et al., 2014; Guo et al., 2016). The underlining reason is that short miRNAs are loaded to AGO1 and 24-nt miRNAs are loaded to AGO4 (Wu et al., 2010a). These two different AGO proteins have high affinities for U starting and A starting miRNAs, respectively (Mi et al., 2008).

Since we obtained large numbers of 24-nt novel miRNAs, we calculated the length distribution of all tomato miRNAs and miRNA stars from this study and the miRBase. We then compared it with those from six other plants (*Physcomitrella patens, Oryza sativa, M. trunc*a*tula, Solanum tuberosum, Nicotiana tabacum*, and *Arabidopsis thaliana*) from different lineages of land plants. Our results showed that the 24-nt miRNAs also accounted the largest proportion in *S. tuberosum* whereas the other four genomes have 21-nt miRNA as the most abundant miRNA type (Figure 2D). This indicates that 24 nt miRNAs may underwent lineage-specific evolution in solanum to remodel its miRNA profile.

### *PHAS* loci in the tomato root and their triggers

**Figure 3:**
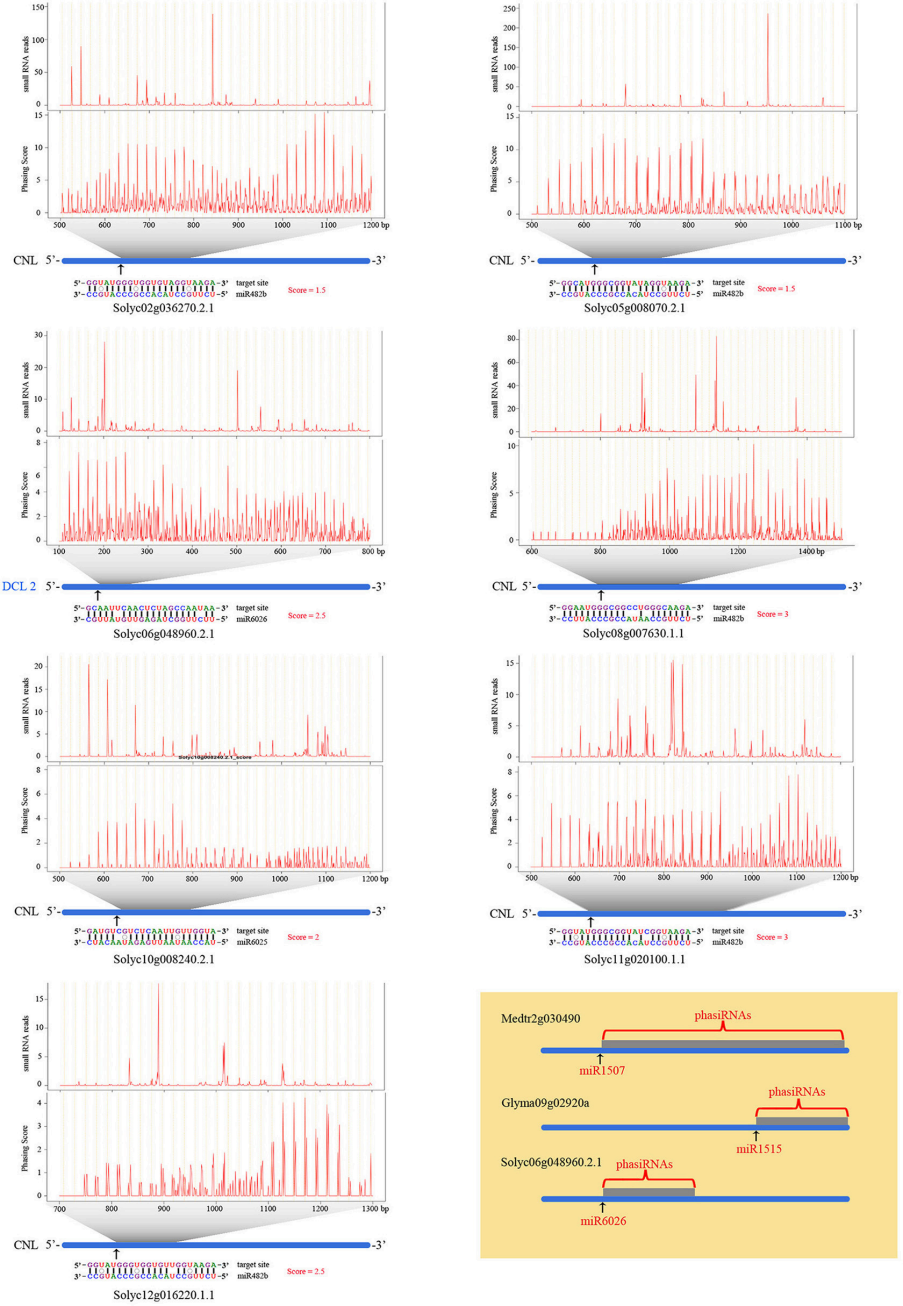
**Seven *PHAS* loci triggered by three 22-nt miRNAs in tomato**. The blue lines are the genes. Below them are alignments of genes and corresponding triggering miRNAs with different nucleotide in different color. In the alignment, vertical lines indicate matches, missing lines indicate mismatches, and circles indicate G:U wobble pairs. The black arrow indicate miRNA target loci. The two figures above the gene shows small RNA read abundance normalized in TPM (transcripts per million) and Phasing score distributions for the region indicated by the gray trapezoids. Horizontal axises of these figures shows the genomic coordinates. The brown yellow figure indicates that different miRNAs target DCL2 among different species.

Plant 21-nt and 22-nt miRNAs can initiate the synthesis of phasiRNAs (phased, secondary small interfering RNAs) by cleaving their target mRNAs into a series of 21-nt siRNAs (Zhai et al., 2011; Fei et al., 2013). From our data, we were able to identify seven *PHAS* loci, which were triggered by three different 22-nt miRNAs (Figure 3). Six such *PHAS* loci were annotated as NBS-LRR type plant disease resistance genes. Five of these NBS-LRR genes were targeted by miR482, while another NBS-LRR gene (Solyc10g008240.2.1) was targeted by miR6025. The only non NBS-LRR *PHAS* gene found in this study was annotated as the tomato *DCL2* and was targeted by miR6026.

### AM-responsive miRNAs in tomato root

**Figure 4:**
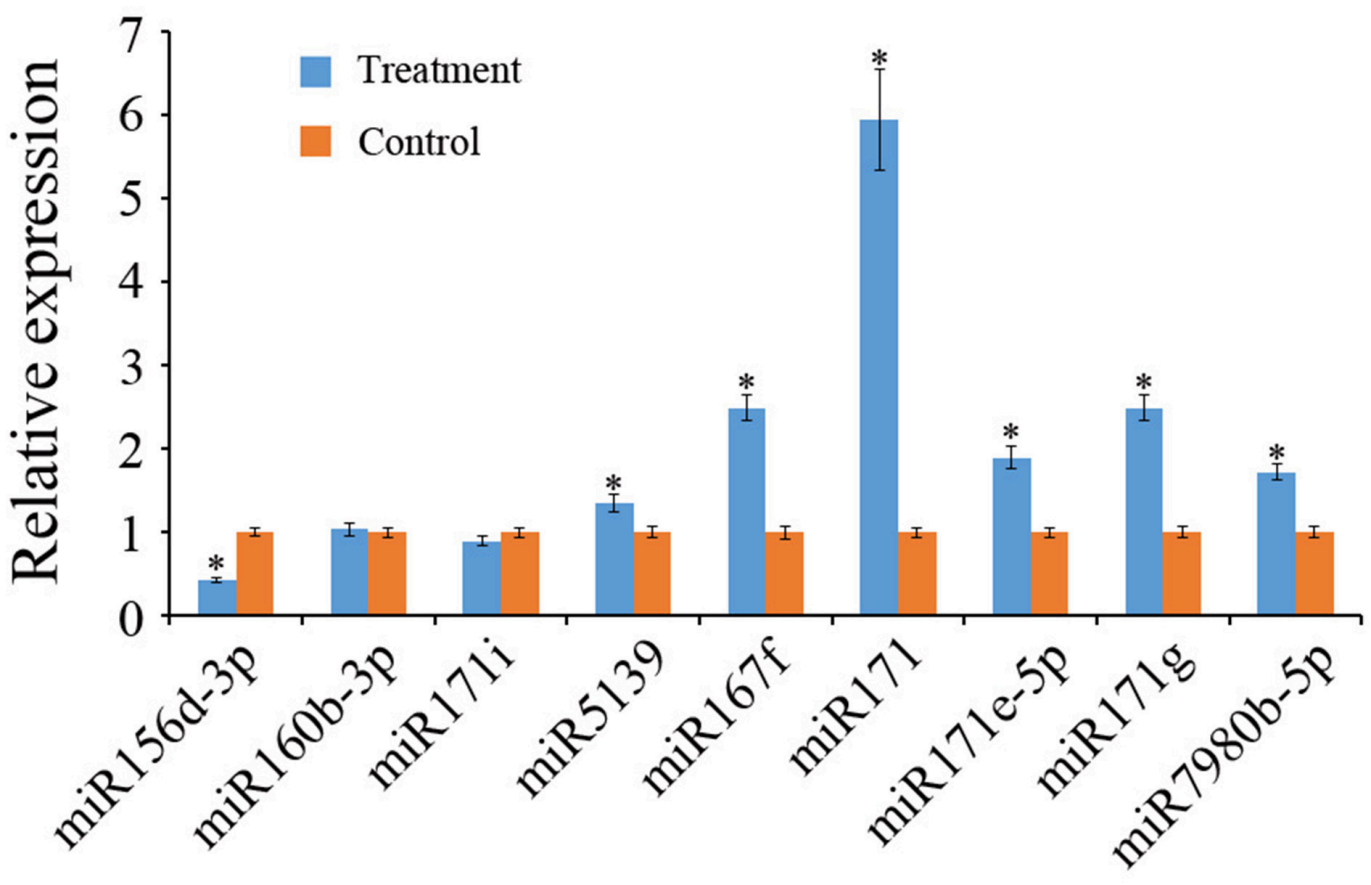
**RT-qPCR validation of expression profiles of nine known miRNAs**. The house-keeping gene U6 was used as internal control, and Error bars represent SEM of three replicates. Asterisks indicate significant difference as determined by Student's *t* test (*P* < 0.05).

Nine known miRNAs in tomato showed a significant difference in read abundance (using a fold-change cutoff of 2) between the treatment and control libraries. Five such miRNAs have significantly more reads and four other ones have significantly fewer reads in the *R. irregularis*-treated sample (Table 3). To confirm the AM responsive expression of these nine miRNAs, we used RT-qPCR to verify their differential expression in the treatment and control samples. Five up-regulated and one down-regulated miRNAs were validated to have a consistent expression pattern with the library sequencing data and they can be deemed as AM-responsive miRNAs (Figure 4). Three of them were miR171 family members, including miR171, miR171g, and miR171i. Interestingly, tomato miR171 and miR171g actually share high sequence identity with a miRNA named Mtr-miR171h, which has been reported to regulate AM symbiosis in *M. truncatula* (Lauressergues et al., 2012; Hofferek et al., 2014). The up-regulation of miR171 and miR171g in tomato revealed by this study, therefore suggests that a conserved miRNA family was adopted by different plant lineages to regulate AM symbiosis.

**Table 3:** **Differentially expressed known miRNAs between treatment and control**.

**MiRNA name**	**Sequence**	**Count (RPM)^a^**		**LFC^b^**	***P*-value**
		**Treatment**	**Control**		
miR156d-3p	GCTCACTGCTCTATCTGTCACC	1.005105	6.354471	−2.66043	1.14E-14
miR160b-3p	GCGTATAAGGAGCCAAGCATA	0.789725	5.640485	−2.8364	5.34E-14
miR171i	TGTTGGAATGGCTCAATCTAA	0.933312	3.998319	−2.09896	1.16E-07
miR5139	AAACCTGGCTCTGATACCA	0.287173	1.427971	−2.31398	0.000955
miR167f	AGATCATGTGGCAGCATCACC	1.005105	1.00E-06	9.97313	5.86E-05
miR171	TTGAGCCGTGCCAATATCACA	9.548497	2.213355	2.109039	1.38E-16
miR171e-5p	AGATATTGATGCGGTTCAATC	0.717932	1.00E-06	9.487704	0.000947
miR171g	TTGAGCCGCGCCAATATCATT	1.220485	1.00E-06	10.25324	7.26E-06
miR7980b-5p	GAGATGAAATCAGCGTTTGGACA	0.717932	1.00E-06	9.487704	0.000947

a *RPM (reads per million: (actual miRNA count/total count of clean reads) × 1000000)*.

b *LFC, log fold change*.

**Figure 5:**
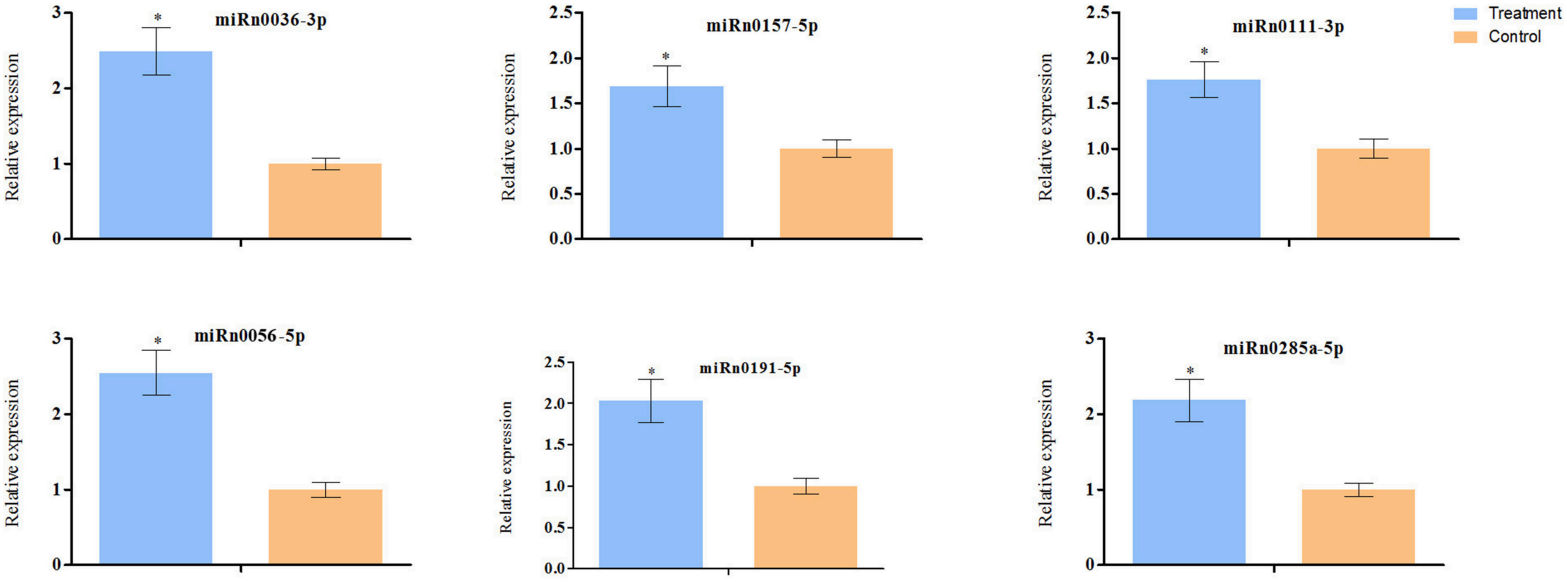
**RT-qPCR validation of expression profiles of six novel miRNAs selected randomly**. The house-keeping gene U6 was used as internal control, and Error bars represent SEM of three replicates. Asterisks indicate significant difference as determined by Student's *t*-test (*P* < 0.05).

**Figure 6:**
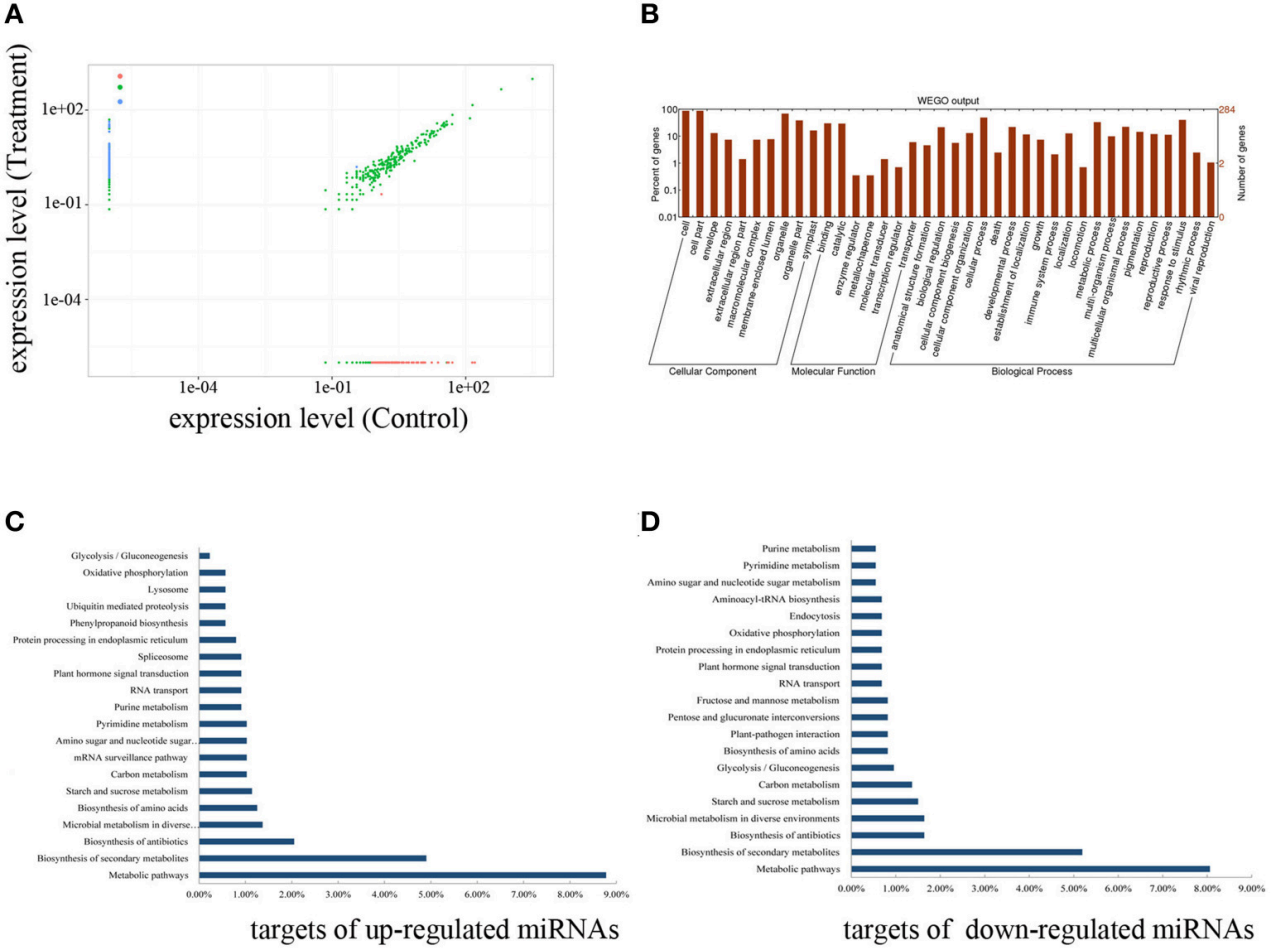
**Differentially expressed novel miRNAs and their target genes. (A)** Each point in the figure represents a novel miRNA. The X axis shows the expression of miRNAs in the control and the Y-axis show the expression of miRNAs in the treatment. Red points represent down-regulated miRNAs (fold-change > 2, *P* < 0.001) and the blue points represent the up-regulated miRNAs (fold-change < −2, *P* < 0.001). **(B)** Go analysis of the novel miRNA target genes. **(C)** Top twenty pathways from KEGG pathway analysis of the target genes of up-regulated novel miRNAs. **(D)** Top twenty pathways from KEGG pathway analysis of the target genes of down-regulated novel miRNAs.

Among the 513 novel miRNAs in tomato, 270 were differentially expressed between the two libraries (using a fold-change cutoff value of 2; *P* < 0.001) including 146 up-regulated (145 were specifically expressed in inoculated roots) and 124 down-regulated miRNAs (Supplementary Table S3). We verified the expression patterns of six miRNAs selected randomly by qPCR and the results coincided with those of the deep sequencing (Figure 5). A previous study in *M. trunctula* revealed that some miRNA stars are induced during AM symbiosis (Devers et al., 2011). In this study, the abundance of 19 miRNA stars was also different between the two libraries under the same criterion (12 up-regulated and 7 down-regulated), which supported the previous study that miRNA stars may also function in gene regulation (Zhang et al., 2011). Taken together, these 289 miRNAs and miRNA stars provide new candidates that potentially regulate AM symbiosis (Figure 6A).

### Target gene prediction of AM-responsive miRNAs

To better understand the regulatory function of the miRNAs during AM symbiosis, the miRNAs that were significantly up- or down-regulated during *R. irregularis* colonization were used to predict their target genes. Using a stringent criterion described in the method, 18 targets for the five known miRNAs were predicted (Table 4). No potential target was predicted for the miR167f under this criterion. For most miRNAs, the predicted number of target genes varied from one to seven. Since all target genes were predicted to be regulated by transcript degradation, we further searched the tomato degradome (http://mirlab.sysu.edu.cn/starscan/Scan.php; Liu et al., 2015) to support the predicted cleavages. The analysis revealed that three of the 18 predicted target genes received the degradome data supported by high *p*-values (Table 4). In *M. trunctula*, one member of miR171 family (miR171h) regulates AM symbiosis by directly targeting the *NSP*2 gene (Lauressergues et al., 2012; Hofferek et al., 2014). Our data revealed that two members of miR171 family (miR171 and miR171g) were predicted to target an *NSP*2 ortholog with low mismatch penalties and also supported by the degradome. This further suggested that some miR171 members might function as common regulators of AM symbiosis in different plant lineages. Besides *NSP*2, miR171 and miR171g have two other common targets, Solyc08g078800.1.1 and Solyc01g090950.2.1. Both of the two genes are annotated as scarecrow-like proteins and their cleavage by miR171 has been validated previously (Huang et al., 2015).

**Table 4:** **Target gene prediction of differentially expressed known miRNAs**.

**MiRNA_name**	**Target_gene**	**Annotation of target gene**	**Expectation**	**Deg^a^**
miR156d-3p	Solyc05g054370.2.1	Acyl-CoA dehydrogenase family	2.5	
miR171	Solyc08g078800.1.1	Scarecrow-like protein 22	1.5	Yes^b^
miR171	Solyc11g013150.1.1	Nodulation-signaling pathway 2 protein	1.5	Yes
miR171	Solyc01g090950.2.1	Scarecrow-like protein 22	1.5	Yes
miR171	Solyc02g085600.1.1	Scarecrow-like protein 15	2.5	
miR171	Solyc03g025700.1.1	Pentatricopeptide repeat-containing protein	2.5	
miR171e-5p	Solyc11g062440.1.1	L-ascorbate oxidase	2.5	
miR171e-5p	Solyc05g008610.2.1	Uncharacterized PKHD-type hydroxylase	2.5	
miR171e-5p	Solyc03g025730.2.1	Tubulin beta-1 chain	2.5	
miR171e-5p	Solyc01g059990.2.1	Serine/threonine-protein phosphatase	3	
miR171e-5p	Solyc01g059980.2.1	Glucan endo-1,3-beta-glucosidase B	3	
miR171g	Solyc08g078800.1.1	Scarecrow-like protein 22	0	Yes
miR171g	Solyc01g090950.2.1	Scarecrow-like protein 22	1.5	Yes
miR171g	Solyc11g013150.1.1	Nodulation-signaling pathway 2 protein	2	Yes
miR7980b-5p	Solyc09g008740.1.1	Uncharacterized	2.5	
miR7980b-5p	Solyc09g008750.1.1	Uncharacterized	2.5	
miR7980b-5p	Solyc09g008760.1.1	Probable cytochrome P450 556A1	2.5	
miR7980b-5p	Solyc06g008310.2.1	Elongator complex protein 2	3	
miR7980b-5p	Solyc06g009430.1.1	Myb-related protein 340-like	3	
miR7980b-5p	Solyc01g096060.2.1	Exportin-2	3	
miR7980b-5p	Solyc06g062780.2.1	Phospholipid-transporting ATPase 6	3	

a *Deg, Degradome analysis results*;

b *Yes, represents the cleavage events were identified in the degradome data (http://mirlab.sysu.edu.cn/starscan/Scan.php; Liu et al., 2015)*.

Using the same method, 1472 and 96 target genes were predicted for the 247 library-specific or differentially expressed novel miRNAs and 16 miRNA star sequences (Supplementary Table S4). We identified 23 of 1472 targets as candidate cleavage targets of novel miRNA in the degradome sequencing data (http://mirlab.sysu.edu.cn/starscan/Scan.php; Supplementary Table S4; Liu et al., 2015). Furthermore, the degradome analysis showed that one target was cleavage by a miRNA star, miRn0398-5p (Supplementary Table S4). The number of potential target genes for each miRNA varied from 1 to 31. Most miRNAs have 1–10 target genes, and one gene can be targeted by several miRNAs at different regions.

All targets were then annotated using Gene Ontology (http://www.Geneontology.org/). GO analysis contains three ontologies, biological process, cellular component, and molecular function. We annotated 316 targets and distributed them in 38 categories, of which ten categories were in cellular component, seven in molecular function, and 21 categories in biological process (Figure 6B). The putative target genes in the molecular function category were related to binding, catalytic, enzyme regulator, molecular transducer, transcription regulator, and transporter. In this category, most target genes were related to binding and catalysis. In the biological process, the targets fell into multiple subcategories. We found many targets related to the response to stimulus. Based on the functional analysis, some targets that were homologs to the genes involved in AM symbiosis were found (Supplementary Table S4). For example, Solyc10g044890.1.1, a homologous gene of DMI1 gene, was predicted to be targeted by miRn0404-3p. Also, Solyc09g098410.1.1, a homologous gene of STR2 (Gutjahr et al., 2012), was predicted to be targeted by miRn0326-3p (Supplementary Table S4).

Based on the further analysis of biological pathways, the targets of AM-responsive miRNAs were mapped to 269 pathways. The metabolic and biosynthesis pathways had the largest percentage of genes targeted by upregulated miRNAs (Figure 6C). The pathways of the genes targeted by downregulated miRNAs were mainly metabolic and biosynthesis pathways, followed by Glycolysis/Gluconeogenesis pathway and Plant-pathogen interaction pathways (Figure 6D).

## Discussion

Although tens of thousands of miRNAs have been identified in land plants as documented in miRBase (Kozomara and Griffiths-Jones, 2014), only a small proportion is shared by different species (Chávez Montes et al., 2014) due to species-specific miRNA innovation during plant evolution. The low expression level and spatial and temporal-specific expression of novel miRNAs hinder complete understanding of miRNA diversity in a species (Chávez Montes et al., 2014; Pandey et al., 2014). As compared with the intensively studied model plants, relatively fewer miRNAs are documented in miRBase for tomato (Kozomara and Griffiths-Jones, 2014). Moreover, the miRNA profile in tomato root is not well-explored. In this study, we conducted high-throughput sequencing of tomato root samples and identified 187 known miRNAs belonging to 47 different families and 513 novel miRNAs belonging to 480 different families.

We cataloged tomato miRNAs into different groups based on their sequence length and found that the 24-nt miRNAs were greatly overrepresented and occupied 39% of all tomato miRNAs (Figure 2D). Although 24-nt sRNAs are generally attributed as siRNAs in many previous studies, a recent study reported that some 24-nt sRNAs are not dependent on RDR2, a protein that essential for siRNA production, indicating they are likely produced using the miRNA pathway. Furthermore, all the reported 24-nt miRNAs in this study have passed the same criteria for the prediction of other short miRNAs. Additionally, by performing northern blot analysis, Moxon et al. (2008) have detected both 21 and 24-nt miRNAs in tomato. In comparison, we found the length distribution of tomato is similar to that of *potato*, which also have the most abundant of 24-nt miRNAs. In contrast relatively fewer 24-nt miRNAs (1–19%) have been found in five other land plants (*P. patens, O. sativa, M. trunc*a*tula, N. tabacum*, and *A. thaliana*) (Figure 2D). This indicates that the solanum might have underwent a lineage-specific evolution to expand its 24-nt miRNAs (Vazquez et al., 2008). As mentioned, plants have two distinct miRNA processing pathways (Wu et al., 2010a; Rogers and Chen, 2013). MiRNAs shorter than 24 nt are transcribed by Pol II, spliced by DCL1, and loaded onto AGO1 to cleavage or inhibit mRNA translation (Rogers and Chen, 2013). In parallel, the 24-nt miRNAs are processed differently; they are spliced by DCL3, and then loading on AGO4 to inhibit target gene transcription by methylation the DNA sequences (Vazquez et al., 2008; Chellappan et al., 2010; Wu et al., 2010a). A recent study in tomato showed that inactivation of DCL3 dramatically decreased the abundance of 24-nt sRNAs (Kravchik et al., 2014). Whether DCL3 in tomato underwent specific evolutionary innovations to strengthen their activity in producing 24-nt miRNA in tomato remains to be determined, but the overrepresented 24-nt miRNAs suggested an enhanced role of DNA methylation on gene expression regulation in this species.

Plant miRNAs shorter than 24 nt usually function by cleavage or binding the target mRNA to regulate a specific gene expression (Rogers and Chen, 2013). Some 21-nt and 22-nt miRNAs are capable of amplifying the regulation spectrum through successively digesting their target mRNA into a series of 21-nt phasiRNAs, which further cascade the regulation effect by targeting more genes (Fei et al., 2013). The miR482/2118 superfamily is a conserved phasiRNA trigger that regulates NBS-LRR gene expression across different plants (Zhai et al., 2011; Shivaprasad et al., 2012). NBS-LRR gene is a huge gene family with dozens to hundreds of members per plant genome (Shao et al., 2014; Zhang et al., 2016). While keeping a large number of NBS-LRR genes is beneficial to the plant for defense against different pathogens, maintaining their expression under a pathogen absent environment also results in a fitness cost (Tian et al., 2003). In *M. truncatula* and soybean, 51 and 39 families of miRNAs were predicted to regulate NBS-LRR genes expression (Shao et al., 2014). Several 22-nt miRNA families (miR157, miR2109, and miR2118) further triggered the digestion of *PHAS* loci into a series of sRNAs that target more than 60% NBS-LRR genes in *M. truncatula* genome (Zhai et al., 2011). Therefore, these miRNAs are recognized as master regulators of NBS-LRR genes. Six miR482-triggering NBS-LRR *PHAS* loci were identified in tomato (Shivaprasad et al., 2012). The miRNA-mediated silencing cascade of NBS-LRR genes was suppressed after pathogen infection, which caused the decreased miRNA expression and promoted increased NBS-LRR gene expression for defense (Shivaprasad et al., 2012). Five miR482 triggering and one miR6025 triggering NBS-LRR loci were identified in our dataset, which further supports the phasiRNA based NBS-LRR gene expression in tomato (Shivaprasad et al., 2012). Interestingly, the expression of miR482 is not compromised upon the infection of *R. irregularis*. This avoidance of activation of defense genes may benefit the host plants for the establishment of symbiotic relationship with *R. irregularis*. Apart from the six NBS-LRR *PHAS* loci, we also found another *PHAS* loci annotated as tomato *DCL*2 gene that is targeted by miR6026. A previous study found that the *DCL*2 gene in *M. trunctula* and soybean are also *PHAS* loci (Figure 3), but are targeted by two other miRNAs (Zhai et al., 2011). This indicates that *DCL*2 gene recruits different miRNAs to regulate its expression in different plants.

A collection of proteins have been identified to be common factors in AM symbiosis; however, miRNAs involved in this process have largely been investigated in several legume species (Combier et al., 2006; Boualem et al., 2008; De Luis et al., 2012; Wang et al., 2014; Holt et al., 2015). The direct regulation of miR171 on NPS2 has recently been validated *in vitro* and miR171h can directly influence the AM in *M. trunctula* (Lauressergues et al., 2012; Hofferek et al., 2014). We identified six AM symbiosis responsive known miRNAs in tomato and revealed that two of the miR171 family members, miR171 and miR171g in tomato, are also capable of targeting *NSP*2. These results suggest that miR171 family may serve as a common regulator of AM symbiosis. Besides *NSP*2, tomato miR171 and miR171g have another two common targets, Solyc08g078800.1.1 and Solyc01g090950.2.1, which are both annotated as scarecrow-like proteins. Cleavage of miR171 on scarecrow-like proteins is one of the earliest evidenced miRNA-target interactions in plants (Llave et al., 2002b). In Arabidopsis, scarecrow-like proteins play important roles in root development (Heo et al., 2011). Therefore, whether the regulation of scarecrow-like proteins by miR171 members has a role in AM symbiosis is interesting. Another AM symbiosis responsive miR171 member is miR171e-5p, which has different predicted target gene with the above miR171 members. One of its targets Solyc01g059980.2.1 encodes a glucan endo-1, 3-beta-glucosidase. A previous research reported that glucan endo-1, 3-beta-glucosidase acts synergistically to degrade fungal cell walls and inhibit the growth of pathogenic fungi (Mauch et al., 1988). The predicted down-regulation of this gene by miR171e-5p may prevent the digestion of *R. irregularis* cell wall by its host. Although no target gene was predicted for miR167 under the strict criterion used in this study, miR167 has been evidenced to regulate the expression of auxin response factors expression in tomato by direct cleavage (Liu et al., 2014). In soybean, the miR167 modulates nodulation and lateral root development by targeting auxin response factors (Wang et al., 2015). Therefore, the up-expression of miR167f in *R. irregularis* infected tomato root in our study indicates that it may also have a role in AM-symbiosis. The identification of above AM symbiosis responsive conserved miRNAs in tomato highlights candidates for exploring common roles of miRNAs in AM symbiosis. However, further experimental analysis would help to verify their exact functions. Gu et al found five other miRNAs (sly-miR158, sly-miR399h, sly-miR408a, sly-miR827b, and sly-miR399m) in tomato root were responsive to AM colonization (Gu et al., 2010, 2014). Sly-miR158 and sly-miR408a were not detected in our research, whereas sly-miR399h, sly-miR827b, and sly-miR399m were detected and renamed as miR399b-3p, miR827-3p, and miR399a-3p. While miR827-3p show similar expression level between the treatment and control in our sequencing datas, the reads numbers of miR399b-3p and miR399a-3p in the treatment were two fold than that in the control, but the difference is not significant in criteria described in the materials and methods.

Additionally, we found that 289 novel miRNAs and miRNA stars in tomato were differentially expressed during *R. irregularis* colonization. Formation of AM symbiosis between plant and fungi is a complex and highly regulated process involving sophisticated morphological and physiological changes in the plant (Schmitz and Harrison, 2014). The expression of hundreds to thousands of genes is altered during symbiosis (Guether et al., 2009; Xue et al., 2015). In the present study, 1618 target genes were predicted for differential expression tomato novel miRNAs, and were assigned to a diversity of pathways, indicating the potential roles of novel miRNAs in modulating global gene expression during AM symbiosis.

## Author contributions

JQC, ZQS and BW conceived and designed the research; PW performed experiments, PW, YW and ZQS analyzed the data, CCL, LWL, FFM, XYW, MW, and YYH participated in data analysis., PW draft the manuscript; ZQS, BW, and JQC modified the manuscript; JQC contributed reagents/materials/analysis tools; all authors have read and approved the manuscript for publication.

### Conflict of interest statement

The authors declare that the research was conducted in the absence of any commercial or financial relationships that could be construed as a potential conflict of interest.

## Supplementary material

Supplementary Figure S1 **Mycorrhizal establishment confirmed by morphological assessment and molecular characterization. (A)** Typical AMF structures, stained with black ink, encountered in tomato roots. Intraradical hyphae (blue arrow), vesicles (red arrow) and arbuscules (yellow arrow) were recognized in the treatment but not in the control. The bars stand for 30 μm. **(B)** RT-qPCR analysis of AM marker genes *SlPT4*. The house-keeping gene actin was used as internal control, and Error bars represent SEM of three replicates. Asterisks indicate significant difference as determined by Student's *t*-test (*P* < 0.05).

Supplementary Figure S2 **An overview of sRNAs from tomato root. (A)** Distribution of clean reads between the two libraries with and without *R. irregularis* colonization. **(B)** Length distribution of clean reads in the two libraries. **(C)** Distribution of unique reads between the two libraries with and without *R. irregularis* colonization. **(D)** Length distribution of unique reads in the two libraries.

Supplementary Table S1 **Known miRNAs identified in tomato root**.

Supplementary Table S2 **Novel miRNAs identified in tomato root**.

Supplementary Table S3 **Differentially expressing novel miRNAs in the *R. irregularis* inoculated tomato roots compared with uninoculated root**.

Supplementary Table S4 **Target genes of the differentially expressing novel miRNAs**.

Supplementary Table S5 **List of primers used**.
