# Supplementary figures and images for: Identification of Arbuscular Mycorrhiza (AM)-Responsive microRNAs in Tomato
Source: Front Plant Sci. 2016 Mar 31;7:429. doi: 10.3389/fpls.2016.00429 (PMC4814767; doi:10.3389/fpls.2016.00429)

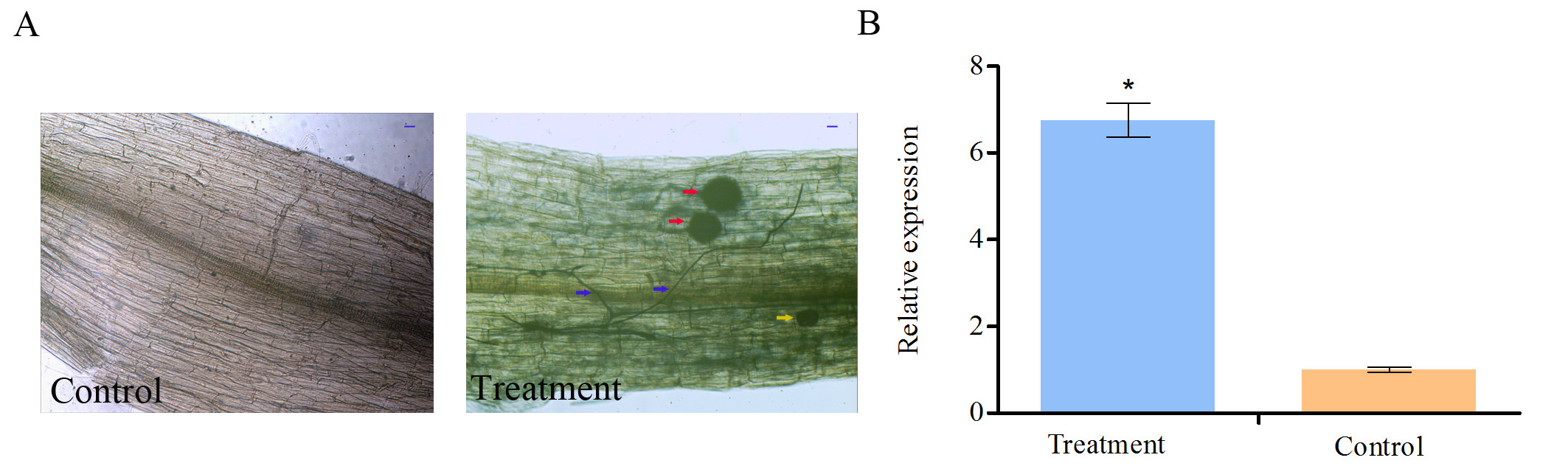

Supplement: Supplementary Figure S1 — Mycorrhizal establishment confirmed by morphological assessment and molecular characterization. (A) Typical AMF structures, stained with black ink, encountered in tomato roots. Intraradical hyphae (blue arrow), vesicles (red arrow) and arbuscules (yellow arrow) were recognized in the treatment but not in the control. The bars stand for 30 μm. (B) RT-qPCR analysis of AM marker genes SlPT4. The house-keeping gene actin was used as internal control, and Error bars represent SEM of three replicates. Asterisks indicate significant difference as determined by Student's t-test (P < 0.05). [file Image1.JPEG]

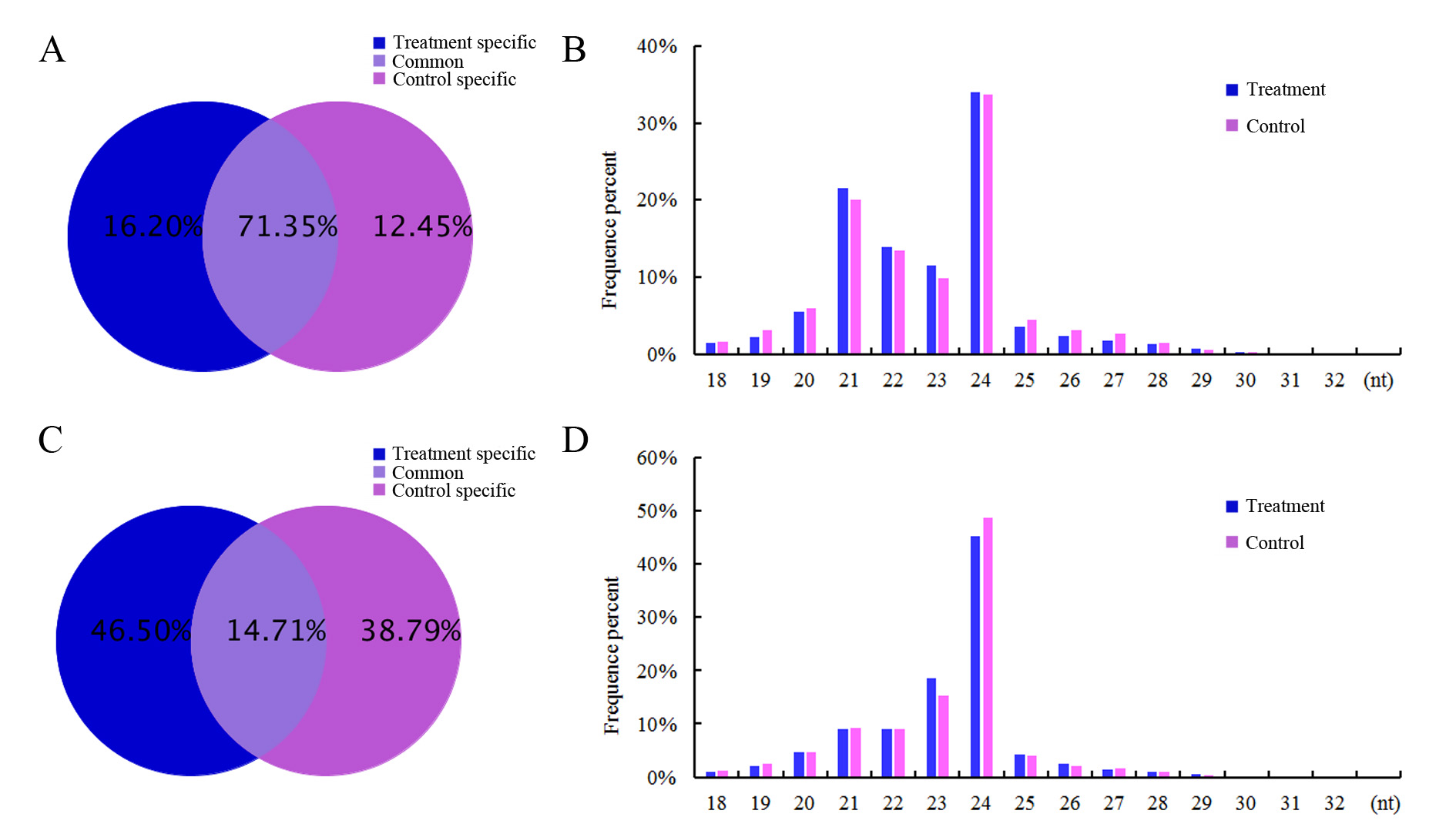

Supplement: Supplementary Figure S2 — An overview of sRNAs from tomato root. (A) Distribution of clean reads between the two libraries with and without R. irregularis colonization. (B) Length distribution of clean reads in the two libraries. (C) Distribution of unique reads between the two libraries with and without R. irregularis colonization. (D) Length distribution of unique reads in the two libraries. [file Image2.JPEG]
